# Thermal stresses in SOFC stacks: the role of mismatch among thermal conductivity of adjacent components

**DOI:** 10.3906/kim-2011-48

**Published:** 2021-03-08

**Authors:** Özgür AYDIN, Go MATSUMOTO, Yusuke SHIRATORI

**Affiliations:** 1 Department of Mechanical Engineering, Faculty of Engineering, Abdullah Gül University, Kayseri, Turkey; 2 Department of Hydrogen Energy Systems, Graduate School of Engineering, Kyushu University, Fukuoka, Japan; 3 International Research Center for Hydrogen Energy, Kyushu University, Fukuoka, Japan; 4 Department of Mechanical Engineering, Faculty of Engineering, Kyushu University, Fukuoka, Japan

**Keywords:** Indirect internal reforming, graded reforming domain, dry reforming, solid oxide fuel cell, thermal analysis, one-cell module

## Abstract

Generating power from renewable biogas in solid oxide fuel cells (SOFCs) is an environment-friendly, efficient, and promising energy conversion process. Biogas can be used in SOFCs via a reforming process for which dry reforming is more suitable as the reforming agent exists in the biogas mixture. Biogas can be directly reformed to *H*_2_-rich fuel stream in the anode chamber of a SOFC by the heat released during power generation. Exploiting the heat and water produced in the SOFC for internal reforming of biogas makes the energy conversion process very efficient; however, various challenges are reported. Thus, indirect internal reforming is opted for which a separate reforming domain is required. In an indirect internal reformer operating at usual conditions, dry reforming rate is quite high in the inlet and it decreases steeply toward the fuel outlet. Great temperature gradients develop over the reformer, since the dry reforming reaction is strongly endothermic. The abruptly varying rate of the reforming reaction affects the temperature fields in the adjacent components of SOFC and hence intolerable thermal stresses emerge on the SOFC components. In our preceding study, we graded the reforming domain, homogenized the temperature profile over the reforming domain, and executed performance and durability experiments. However, most of the experiments failed due to fracturing SOFC components hinting at existence of thermal stresses. In that study, we focused on minimizing the temperature gradients within the reforming domain; namely, we neglected the other processes. To eliminate the thermal stresses, we modeled the entire module of SOFC equipped with a reformer featuring a graded reforming domain. We found that the mismatch between the thermal conductivities of the adjacent module components is the major reason for the thermal stresses. When the mismatch is eliminated, thermal stresses disappear even if the reforming domain is not graded.

## 1. Introduction

For the environment-friendly power generation by fuel cells, *H*_2_ is widely considered as a suitable energy carrier. State-of-the-art fuel cells exhibit rather promising electrochemical performances when they are fed by *H*_2_ . However, the infrastructure for storing/distributing *H*_2_ is not sufficient yet. Thus, the consumers are very much worried about obtaining *H*_2_ , even though they are highly enthusiastic for employing fuel cells. In other words, the insufficient infrastructure of *H*_2_ is standing as one of the major obstacles before the spread of fuel cells. In this regard, hydrocarbon fuels (*CH*_4_ , *C*_4_*H*_10_ , etc.) are more advantageous. Owing to the common use of hydrocarbons, the infrastructure for storing and distributing these fuels has been sufficiently developed [1]. Therefore, on-site production of *H*_2_ from hydrocarbons is widely accepted as a promising solution. Another fact that supports on-site production of *H*_2_ from hydrocarbons is that, currently a huge portion of *H*_2_ is extracted from hydrocarbons. Of course, this concept does not fully comply with the environment-friendly power generation by *H*_2_ fuel cells. Nonetheless, it can increase the efficiency of the power generation in comparison to the conventional technologies, as fuel cells are rather efficient energy converters. Thus, on-site production of *H*_2_ from hydrocarbons is more advantageous in terms of the environmental concerns. Until the infrastructure for storing and distributing *H*_2_ becomes satisfactory, on-site production of *H*_2_ from hydrocarbons will be very useful. In order to approximate the environment-friendly power generation, biosources can be considered as the renewable source of hydrocarbons [2, 3, 4]. Particularly, biogas with its typical composition of 60% *CH*_4_ and 40% *CO*_2_ is broadly regarded as a reliable feedstock for fuel cells [5, 6, 7].

According to the thermodynamic considerations, the direct electrochemical oxidation of *CH*_4_ (in biogas) is a quite efficient way of power generation due to the small entropy change [8]. However, despite the constant interest and extensive investigations, a satisfactory fuel cell performance with direct oxidation of *CH*_4_ is still expected [9]. Thus, currently hydrocarbons are utilized in fuel cells upon a suitable reforming process. Regarding the reforming processes, we can say that dry reforming (DR) is more advantageous for especially biogas due to the fact that the reforming agent *CO*_2_ inherently exists in the biogas. For other reforming processes, e.g., steam reforming, partial oxidation, etc., the reforming agent must be provided from an external source by an appropriate device at a certain cost. Therefore, DR seems more advantageous for fuel cell systems utilizing biogas.

For producing *H*_2_ from biogas via DR, an external reactor can be employed. DR in a reactor requires an external supply of thermal energy at a particular cost and complexity [10, 11]. However, in regard of reforming biogas for fuel cells there is an interesting fact. In fuel cells, chemical energy of *H*_2_ is partially converted to thermal energy, albeit the primary goal is to generate electric power. Use of this thermal energy is another field of interest in designing fuel cell systems. Indeed, the thermal energy required for producing *H*_2_ via the DR reaction can be provided by the fuel cells which utilize the produced *H*_2_ . Coupling fuel cells with the reactors serving for the DR process also helps for cooling the cells without an external supply of cooling air. Elimination of the external heat and air supply enhances the overall efficiency of the system (electric and thermal) significantly. It also makes the on-site utilization of renewable biogas in the fuel cell systems more advantageous.

Regarding the on-site DR process, effective transfer of thermal energy released in fuel cells to the reactor(s) is quite important. In this regard, considerations in terms of heat transfer should be taken into account. From the chemistry point of view, DR, water-gas shift (WGS), and electrochemical hydrogen oxidation (EHO) reactions can proceed simultaneously in the same domain as long as the reaction conditions are met. The anode of a solid oxide fuel cell (SOFC) is indeed a good domain for the aforementioned reactions to proceed in parallel, as it meets all the requirements. When DR takes place in the anode, all the worries about the heat transfer disappears; because the thermal energy is consumed directly where it is released. Due to the direct characteristic of DR and the associated heat transfer, these systems are called direct internal reforming SOFCs (DIR-SOFCs). In terms of the overall efficiency of fuel cell systems, DIR-SOFCs approximate the fuel cells in which *CH*_4_ is directly oxidized: the thermal energy and *H*_2_*O* released from the EHO reaction are directly used in the reforming reactions. Also, in terms of the challenges, DIR-SOFCs resemble the systems where *CH*_4_ is directly oxidized. Hence, realization of DIR-SOFCs is still doubted due to various problems, e.g., carbon deposition, *H*_2_*S* poisoning, heterogeneous cooling, etc. [2, 12, 13, 14, 15, 16].

The challenges associated with DIR-SOFCs can be resolved to an extent when we design a separate reforming domain that can absorb the thermal energy from the SOFC domain. This concept is referred to as indirect internal reforming SOFCs (IIR-SOFC). In terms of the overall system efficiency, IIR-SOFCs are as efficient as DIR-SOFCs, since the thermal energy is supplied from the SOFC domain, and the SOFC domain is cooled down without an external supply of air. Besides, *H*_2_*O* released from the EHO reaction can join reforming of *CH*_4_ via steam reforming and WGS reactions. Thus, the negative impact of excess *H*_2_*O* on the open-circuit voltage (OCV) of the SOFC domain can be alleviated, as in the case of DIR-SOFCs. Another advantage of IIR-SOFCs is that the produced *CO* and the remaining amount of *CH*_4_ after the reformer can be reformed in the anode, as *H*_2_*O* is available at plenty amounts.

Even if indirect internal reforming increases the cost of SOFC systems in comparison to the DIR-SOFCs, it allows for overcoming the most of the challenges and big temperature gradients remain as the major issue in IIR-SOFCs [10, 3, 17, 14, 18, 15, 19]. The temperature gradients are consequences of the drop in the rate of endothermic DR reaction which is due to the decrease in the partial pressures of the reacting specie across the reforming domain. At usual operating temperatures of SOFCs, the rate of the reforming reaction is rather high. When the reforming domain is designed homogeneous in terms of catalytic activity, the reforming process happens very quickly and it almost terminates in the inlet vicinity of the reforming domain [10, 3, 17, 14, 18, 15, 19]. The rest of the reforming domain remains relatively passive with negligible contribution to the reforming process. As a result, steep temperature gradients emerge particularly in the inlet region [10, 3, 17, 14, 18, 15, 19]. Since an effective transfer of the thermal energy from the SOFC domain to the reforming domain is essential in IIR-SOFCs, the thermal balance in the reforming domain directly affects that of the SOFC domain, i.e. steep temperature gradients develop in the SOFC domain as well. Hence, the PEN (positive-electrolyte-negative) components are exposed to high thermal stresses.

As explained, the thermal stresses are directly linked to the steep drop in the reforming rate. The drop can be alleviated by controlling the reforming rate via grading the reforming domain in terms of catalyst loading. A reforming domain can be graded with the segmentation method in which the reforming domain consists of segments (subdomains) fabricated with proper catalyst loading. In this regard, a number of studies have been carried out as summarized in the following. Nagata et al. carried out a numerical study on a tubular SOFC which was equipped with a prereformer and an indirect internal reformer [10]. By grading the reforming domain in terms of catalyst loading in the indirect internal reformer, they reduced the temperature gradients in the SOFC domain. Similarly, Nishino et al. conducted a numerical study on a tubular IIR-SOFC and they also reported that the temperature gradients in the SOFC domain can be mitigated by adjusting the catalyst loading [20]. Shiratori et al. realized a graded reforming domain and experimentally demonstrated the feasibility of the method [3]. In a relevant study, Pajak et al. conducted a numerical study on a reactor [21]. It was reported that they significantly improved the temperature distribution along the reactor by grading the catalyst loading. In the same year, we conducted extensive experimental and numerical studies on a standalone reformer to develop a reliable numerical tool for designing graded reforming domains to be employed in IIR-SOFC stacks [19]. In that project, we managed to derive a relation between the preexponential coefficient of the rate equation (DR reaction) and catalyst loading. By using this relation, we designed a graded reforming domain for a homogenized temperature profile and we further experimentally demonstrated the computed temperature profile. We continued with another study where we equipped a one-cell module of SOFC with an indirect internal reformer [22]. In that study, we designed and fabricated the indirect internal reformer with a graded reforming domain. Afterwards, we integrated the reformer exhibiting a homogenized temperature profile to the one-cell module of SOFC. On this IIR-SOFC module, we carried out electrochemical performance and durability tests along with the temperature measurements. Most recently, Serincan et al. reported a similar approach for controlling the reforming rate to alleviate temperature gradients [23]. They reported that reducing the catalytic activity of anode by 1/100 allows for more homogeneous temperature profile.

In the study of the one-cell module of IIR-SOFC, we initially investigated the temperature variations across the thickness of the reformer with the help of a numerical model [22]. It was observed that the temperature variations significantly reduced toward the top and bottom covers of the reformer. Leaning upon this finding, it was interpreted that the effect of the endothermic reforming reaction on the temperature field in the SOFC domain should be small. Particularly when the reforming domain was graded and the temperature gradients in the reforming domain was reduced significantly, it was thought that the impact of the endothermic reaction on the temperature field of the SOFC domain would be negligible. Thus, an indirect internal reformer with a graded reforming domain was directly attached to the one-cell module of SOFC. Then, trials for electrochemical performance and durability tests were done. However, a number of experiments failed; the performance and durability tests were hardly completed due to fracturing SOFC domains. The fractures in the SOFC domains were pointing to thermal stresses and underlying temperature gradients. As the reformer was already analyzed deeply in terms of the thermal balance, thermal analysis of the entire one-cell module of SOFC with the reformer was required. Thus, we devoted this study to the thermal analysis of the module, for which we modeled the entire module by incorporating the previously overlooked processes.

This study is distinguished from the related studies due to the following aspects:

- It is the first study presenting a complete 3D model of a one-cell module of SOFC equipped with an indirect internal reformer featuring a graded reforming domain.
- For the first time it reveals the importance of the mismatch among thermal conductivity of adjacent module components for preventing thermal stresses over the components of internal reforming SOFCs.
- It proves that the graded the reforming domain which has been considered as a potent solution for minimizing thermal stresses in internal reforming SOFCs cannot minimize the temperature gradients.

## 2. Numerical model

Designing a graded reforming domain on a module/stack of IIR-SOFC is a big challenge in terms of the numerical computations. Thus, in our preceding study we designed the graded reforming domain on a standalone reformer and then we integrated the reformer into the one-cell module of IIR-SOFC [22]. We adopted this simple approach leaning upon the fact that the endothermic reforming reaction is the most dominant in terms of the thermal balance in the IIR-SOFC module. However, the experimental failures during the performance and durability tests of the IIR-SOFC module hinted at intolerable thermal stresses on the PEN components despite the graded reforming domain. Therefore, in this study we tried to model the entire module for an extensive thermal analysis that would reveal crucial points for us to avoid thermal stresses in the PEN components. Properties of the module is explained in detail in the preceding study [22].

**Figure 1:**
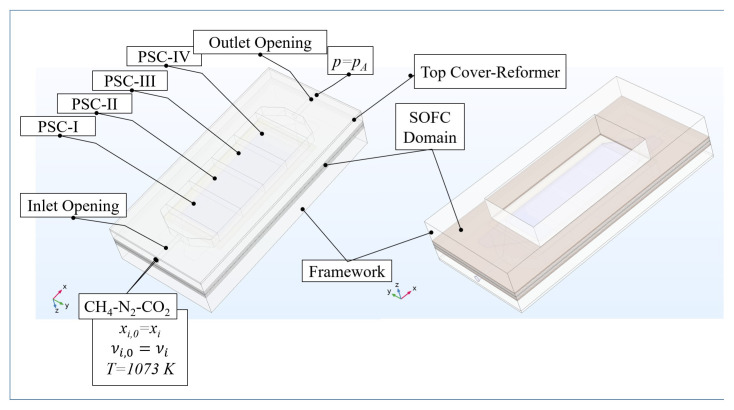
The computational domain of the IIR-SOFC module on which FEM was applied for the mass, momentum, charge, and energy balances.

**Figure 2:**
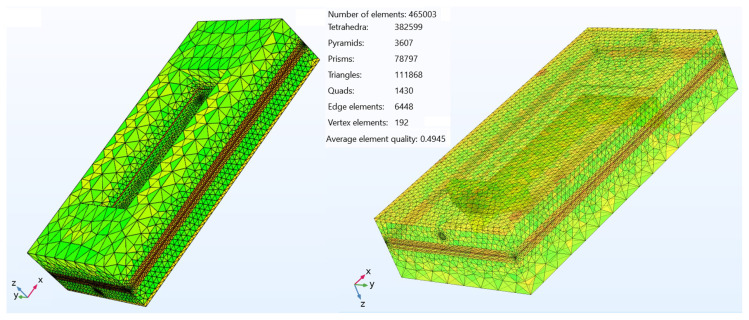
Meshed computational domain of the IIR-SOFC module and mesh statistics.

We modeled all the involving processes of mass, momentum, charge, and energy transfer in three dimension in COMSOL Multiphysics by applying finite element method (FEM) on the computational domain illustrated in Figures 1 and 2. We assumed that i) the system was steady, ii) the chemical specie were ideal gases, and iii) the module components possessed isotropic microstructures.

### 2.1. Mass balance

Mass transport processes in the reformer, anode, and cathode were modeled separately. In the reformer *CH*_4_*,CO*_2_*,H*_2_*,N*_2_ , in the anode *H*_2_*,N*_2_*,H*_2_*O*, and in the cathode *O*_2_*,N*_2_ were taken into account. Properties of these gases are presented in Tables 1–3.

For modeling the mass transport in the free-flow and porous domains (Figure 1), we applied the Stefan–Maxwell equation


(1) $$\nabla \cdot j_{i}+\rho (u\cdot \nabla )w_{i}=S_{i}\;\;\;i\in (CH_{4},CO_{2},CO,H_{2},N_{2},H_{2}O)$$

where *u* (*m s*^−1^) and *ρ* (*kg m*^−3^) indicate the velocity and density, respectively. *w_i_* , *S_i_* , and *j_i_* denote the mass fraction, source term, and diffusive mass flux vector of species *i* , respectively. The velocity was computed in the momentum balance (subsection 2.2). The density was computed by


(2) $$\rho =\frac{pM_{i}}{RT},$$

where *p*(*Pa*) , *M_i_*(*kg/mol*) , *R*(*J/molK*) , and *T*(*K*) stand for the pressure, molar mass, gas constant, and temperature, respectively. The pressure was calculated in the momentum balance, while a constant temperature of 1073 K was considered in the computations.

The mass fraction was provided as an inflow boundary condition (Figure 1). In the real one-cell module of IIR-SOFC, the reaction products leaving the reformer directly go into the anode of the SOFC domain [22]. However, this flow pathway was split in the numerical model such that, the *H*_2_*,N*_2_ equivalent of the mixture supplied to the reformer was provided as the inlet boundary condition of the anode.

The source term in Eq. 1 was employed for taking account of the mass conversion in the reforming and SOFC domains. For modeling the reforming process, the dry reforming reaction was considered. Detailed information about the model of dry reforming is provided elsewhere [19].

For simplifying the model, the mass conversion in the cathode due to the EHO reaction was ignored owing to the air supply at excess amounts. However, the mass conversion in the anode was modeled by using the source term (*S_i_* ) in Eq. 1 as described in detail elsewhere [25].

The diffusive mass flux vector in Eq. 1 is


(3) $$j_{i}=-\rho w_{i}\sum_{j}D_{ij}d_{j}-D_{i}^{T}\frac{\nabla T}{T},$$

where $D_{i}^{T}(m^{2}s^{-1})$, *D_ij_* (*m*^2^*s*^−1^) , and *d_j_* indicate the thermal diffusion coefficient, binary diffusion coefficient of the species *i* and *j* , and diffusion driving force, respectively. The binary diffusion coefficient was calculated based on the kinetic gas theory [26], as explained elsewhere [25].

### 2.2. Momentum transfer

For calculating the velocity and pressure fields in the free-flow and porous domains, the Navier–Stokes equation and the Brinkman equation were applied respectively along with the continuity equation. Comprehensive information about the momentum balance is provided elsewhere [25].

### 2.3. Charge balance

In fuel cells, electrochemical, ionic, and electronic charge transfer processes take place. The electrochemical charge transfer was modeled by the Butler–Volmer equation, while the ionic and electronic charge transfers were modeled by the Ohm’s law. Regarding properties are provided in Table 4 and the details of the charge transfer processes are explained elsewhere [25].

### 2.4. Heat balance

The thermal balance in the one-cell module of IIR-SOFC involves production and consumption of heat in the SOFC and reforming domains, respectively; transfer of heat via conduction, convection, and radiation in the regarding domains or surfaces. Thermo-physical properties of the module components are presented in Table 5.

In order to model all the involving processes, the general heat balance equation was applied along with the Stefan–Boltzman law. The heat balance equation was formulated as


(4) $$\rho C_{p,i}u\cdot \nabla T=\nabla \cdot (\lambda_{m}\nabla T)+Q_{h},$$

where *C_p,i_* (*J kg*^−1^*K*^−1^) , *λ_m_* (*W m*^−1^*K*^−1^) , and *Q_h_* (*W m*^−3^) show the specific heat, thermal conductivity, and source/sink term. The density and velocity were linked to the mass (subsection 2.1) and the momentum (subsection 2.2) balance equations, respectively. The specific heat and thermal conductivity of the gas mixtures flowing in the reformer, anode, cathode were defined as presented elsewhere [25]. Heat transfer via radiation was modeled according to the definition given elsewhere [19]. For considering the thermal energy consumed in the reforming domain, and produced in the SOFC domain, *Q_h_* in Eq. 4 was employed. The heat consumption was modeled according to the description provided in [19]. The heat production was modeled as


(5) $$Q_{h}=(\frac{\Delta H_{EHO}}{n_{s}F}-\Phi_{cell})i,$$

where Δ*H_EHO_* (*kJ/mol*) , *n_s_* , *F* (*C/mol*) , Φ*_cell_* (*V*) , and *i* (*Acm*^−2^) indicate the enthalpy change of the EHO reaction, number of electrons participating in the reaction, Faraday’s constant, operating cell voltage, and current density, respectively.

## 3. Results and discussion

Regarding the fracture of the PEN components occurred during the performance and durability tests of the one-cell module of IIR-SOFC [22], we suspected on the thermal stresses and associated temperature gradients over the PEN components. Since we graded the reforming domain, we were sure that the temperature gradients were significantly reduced in the reformer. This was supported by the real-time temperature profiles in situ measured along the paper-structured catalyst (PSC) [19] segments during the experiments. Therefore, it was thought that the reason for the fracture of the PEN components could not be the steeply dropping rate of the endothermic reforming reaction. In order to clarify the phenomenon, we decided to carry out a thermal analysis of the IIR-SOFC module numerically. For the analysis, we considered the operating conditions of the module during the durability tests [22], as the PEN components were broken during these investigations.

### 3.1. Model validation

For ensuring accuracy and reliability of the numerical model developed, we carried out two distinct validation studies where all the processes occurring in the IIR-SOFC module were incorporated. One of the validation studies was conducted for the charge balance in the SOFC domain, while the other one was executed for the thermal balance in the entire module. For verifying the model in terms of the charge balance, the in situ obtained polarization curve of the module was employed [22]. To validate the model in terms of the thermal balance, temperature data in situ acquired along the PSC segments were considered [22]. Due to the fact that the extent of the temperature variation in the reformer because of the endothermic reforming reaction is rather small to be effective on the charge transfer processes, the model was initially validated in terms of the charge balance.

**Figure 3:**
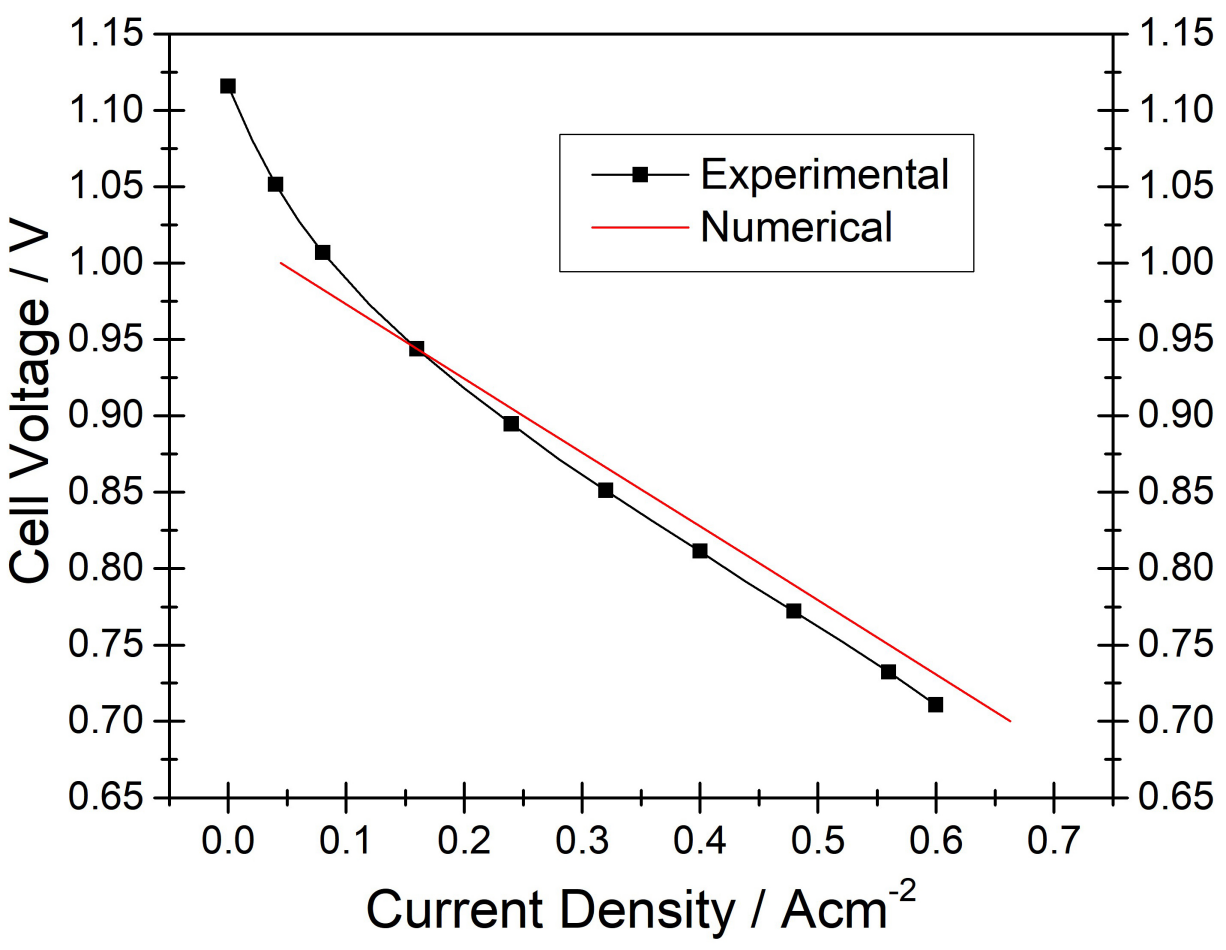
Experimentally and numerically obtained polarization curves of the SOFC domain compared for *CH*_4_*/CO*_2_*/N*_2_ = 40*/*40*/*100 *ccm*. Note that the reforming domain was graded with 0.088, 0.176, 0.396, 0.88 *wt*% nickel and all the processes occurring in the module were taken into account.

Figure 3 shows the experimental and numerical polarization curves. We observe that the model captures the in situ acquired polarization curve with rather small deviations. This good correlation allows us to interpret that the heat production in the SOFC domain and its impact on the thermal balance of the IIR-SOFC module can be accurately computed.

**Figure 4:**
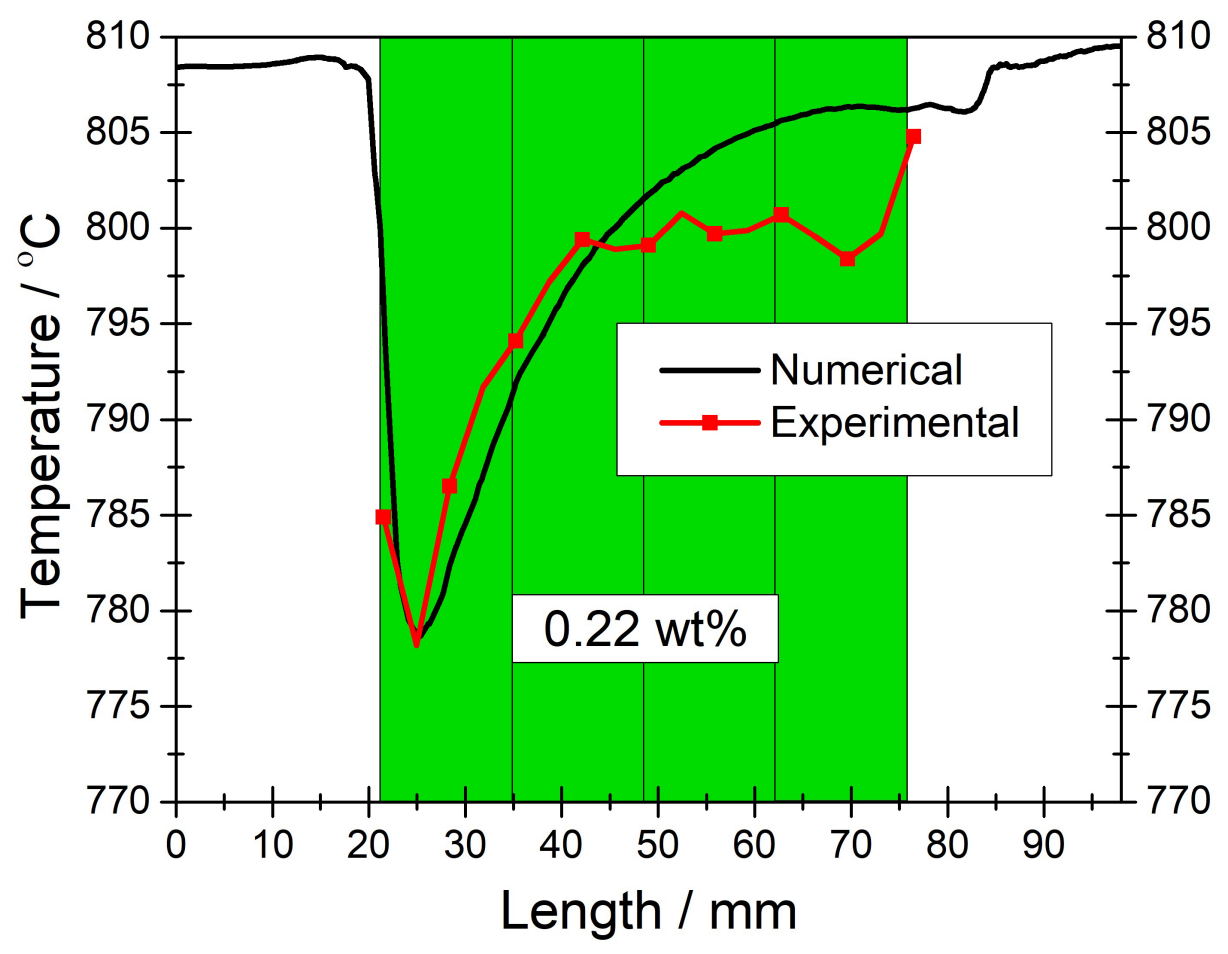
Experimental and numerical temperature profiles obtained in the middle of the reforming domain (y = 24 mm) in the flow direction for *CH*_4_*/CO*_2_*/N*_2_ = 40*/*40*/*100 *ccm* and 0.3 *Acm*^−2^ . Note that the reforming domain was homogeneous with an identical nickel loading of 0.22 *wt*%.

In Figure 4, experimental and numerical temperature profiles are plotted along the reformer where the PSC segments accommodated an identical nickel loading of 0.22 wt%. It should be noted that 0.22 wt% nickel was determined as the minimum catalyst loading that is required for achieving sufficient *CH*_4_ conversion in the reformer. With a uniform catalytic activity, the temperature profile along the reformer was rather heterogeneous with a quite steep drop in the inlet region of the PSC segments. Since the temperature was measured along the PSC segments, only numerical data were available outside the PSCs segments. When the experimental and numerical temperature profiles are compared, a good correlation is observed between them; the deviations can be attributed to the imprecision in positioning the thermocouples during the experiments [19, 22]. From this finding, we understand that the temperature computations were accurate and hence we could use this numerical model for calculating the temperature profile along the PSC segments when they were graded in terms of the catalyst loading. Besides, the model can be used for designing a different graded reforming domain for minimizing the temperature gradients in the PEN components of the module.

**Figure 5:**
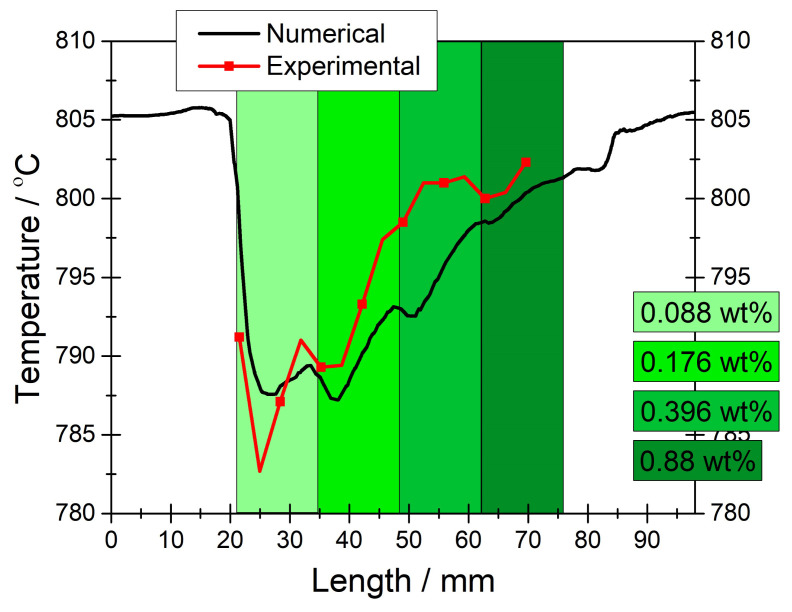
Experimentally and numerically obtained temperature profiles in the flow direction along the graded (0.088, 0.176, 0.396, 0.88 *wt*% nickel) reforming domain for *CH*_4_*/CO*_2_*/N*_2_ = 40*/*40*/*100 *ccm* and 0.3 *Acm*^−2^ .

Figure 5 displays the numerical and experiential temperature profiles along the reformer for a graded reforming domain of 0.088, 0.176, 0.396, 0.88 wt% nickel from inlet to outlet, respectively. It should be pointed out that the nickel amounts in the PSC segments are exactly the same as designed on the standalone reformer in our preceding study [22]. As stated, due to the fact that temperature was measured along the PSC segments, only numerical data available in the remaining part of the reformer. As the numerical and experimental temperature profiles are contrasted, a rather good correlation is observed between them. Again, the deviations can be attributed to the imprecision in positioning the thermocouples during the experiments [19, 22].

### 3.2. Temperature profiles along module components with graded reforming domain

**Figure 6:**
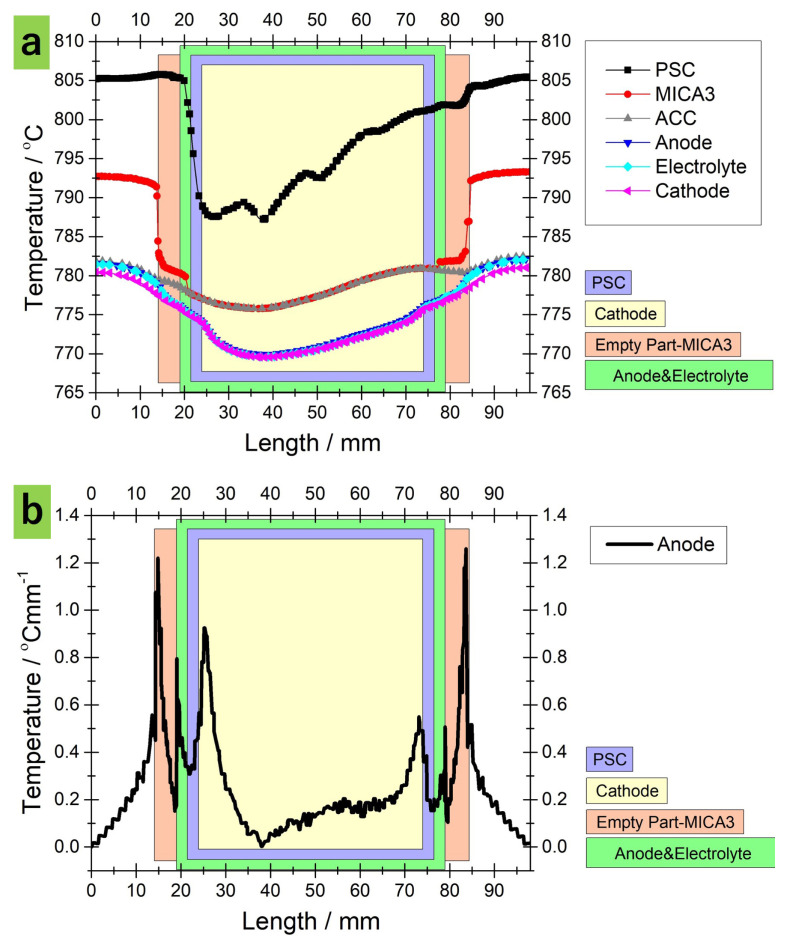
For 0.3 *Acm*^−2^ and *CH*_4_*/CO*_2_*/N*_2_ = 40*/*40*/*100 *ccm* a) numerical temperature profiles extracted in the middle of the reforming domain (y = 24 mm) in the flow direction over various module components; b) temperature gradient (dT/dx) along the anode. Note that the reforming domain was graded with 0.088, 0.176, 0.396, 0.88 *wt*% nickel, as designed in [22].

Figure 6 presents temperature profiles over various components of the IIR-SOFC module and the temperature gradient (dT/dx) over the anode. When we focus on the reforming domain, a steep temperature-drop is still observed in the inlet region even if a smoother temperature-rise appears toward the outlet. These steep and smooth temperature changes in the inlet and outlet of the reformer can be ascribed to the design of the reforming domain in terms of the catalyst loading as well as to the thermal conductivity of the materials in the regarding components of the module. In other words, temperature would not drop in the reforming domain if the chemical species in the flow and the PSC segments had thermal conductivities approaching to that of the reformer frame, as is seen in the case of anode current collector (ACC).

We see in Figure 6a that the temperature profiles along MICA3 and ACC overlap except the inlet and outlet regions of the reformer. While MICA3 exhibits steep temperature gradients in the inlet and outlet regions, ACC shows rather smooth transitions. The steep temperature gradients in the temperature profile of MICA3 are due to the low thermal conductivity of the chemical species present. The geometry of MICA3 was like a frame, in which there were free volumes in the inlet and outlet regions and the PSC segments occupied the remaining free space. Owing to the high thermal conductivity of Crofer, the impact of the endothermic reforming reaction on the temperature profile of the ACC is quite small. In the case of the reformer, the temperature profile stands for different materials of the reformer-frame, fluids, and PSC segments, where thermal conductivity of the fluids and PSC segments are relatively low. It is interesting to note that the temperature profiles in MICA3 and ACC are rather smooth despite the variations of the temperature profile in the reformer. This indicates that the higher thermal conductivity of ACC prevails on the thermal balance of the components, so that the heterogeneous feature of the reforming reaction along the reformer cannot dominate on the temperature profile of the adjacent components. It should be remarked that this finding was the basis for focusing onto the reformer’s thermal balance in the preceding study of one-cell module of IIR-SOFC [22].

Temperature profiles in the anode, electrolyte, and cathode of the module are also displayed in Figure 6a. First of all, the temperature profile of the PEN components are almost the same. These similar temperature profiles are attributed to the facts that the thermal conductivity of these components are very close to each other (Table 5) and these components have almost the same thickness [22]. Alike the temperature profile in the reformer, temperature profiles of the anode, electrolyte, and cathode display steep changes particularly in the inlet region. Similar to the reforming domain, the PEN components were surrounded by a high thermal conductivity material of Crofer (Table 5) in which heat was easily conducted. However, the low thermal conductivities of the PEN components do not allow heat to spread toward the inner regions and hence rather steep temperature gradients occur in the inlet region. From these findings we understand that the endothermic reforming reaction dominates on the temperature profiles of the PEN components.

Regarding the thermal stresses and temperature gradients in the internal reforming SOFCs, Figure 6 reveals a very crucial evidence. While temperature profiles of the PEN components and reforming domain approximate with some deviations along the cathode, they are distinguished in terms of the distance along which the endothermic reforming reaction is effective. In the reformer, temperature starts to fall rapidly at ca. 21 mm and it recovers around 76 mm. The distance at which the temperature in the reformer falls quickly is the point where the reforming process begins; the point at which the temperature in the reformer recovers is approximately where the reforming process terminates. Namely, the temperature profile in the reformer exactly indicates the region where the reforming process occurs. However, temperature in the PEN components begins to drop steeply around 24 mm and it recovers at ca. 74 mm. Thus, the respective temperature gradients peaks around 24 mm and 74 mm as illustrated in Figure 6b. The distance between the points at which the temperature in the PEN components steeply drop and rise corresponds to the length of the cathode. This indicates that the endothermic reforming reaction was dominant only along the cathode, albeit it occurred along the PSC segments. The reason for the PEN components not to experience the prevailing impact of the endothermic reforming reaction along the PSC segments is that the PEN components were surrounded by a metal spacer of Crofer [22] which has a rather high thermal conductivity. Despite the larger dimensions of the anode and electrolyte comparing to the cathode, they also do not exhibit steep temperature slops in the region where the reforming process occurs due to the thermally conductive metal spacer around the cathode.

### 3.3. Importance of thermal conductivity

For investigating the role of thermal conductivity of the components adjacent in the module, we carried out a different numerical study. To make the PEN components and the adjacent spacer have close thermal conductivities, we changed the material of the Spacer-2 from Crofer to MICA [22]. With this model, we executed computations for the graded and uniform reforming domains separately.

**Figure 7:**
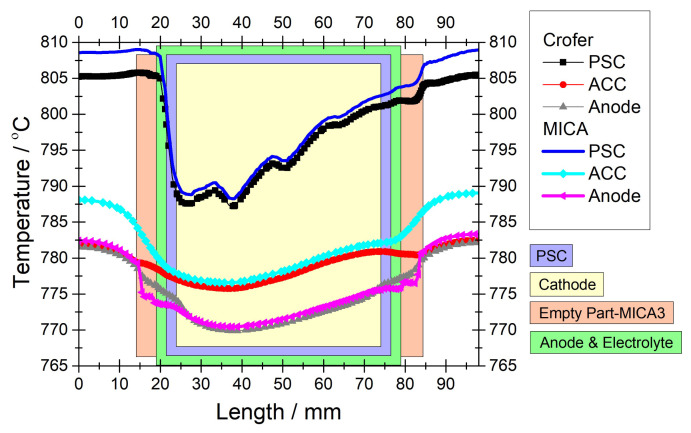
Numerical temperature profiles over various module components compared with respect to the material of the Spacer-2. Note that the data were extracted in the middle of the reforming domain (y = 24 mm) in the flow direction for 0.3 *Acm*^−2^ and *CH*_4_*/CO*_2_*/N*_2_ = 40*/*40*/*100 *ccm*. The reforming domain was graded with 0.088, 0.176, 0.396, 0.88 *wt*% nickel as designed in [22].

**Figure 8:**
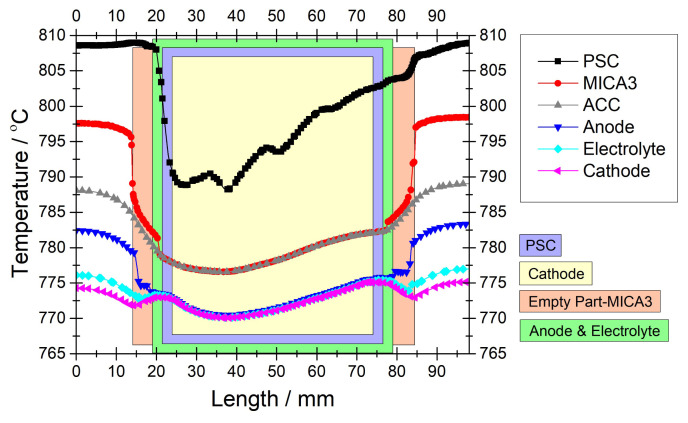
Numerical temperature profiles extracted in the middle of the reforming domain (y = 24 mm) in the flow direction over various module components for 0.3 *Acm*^−2^ and *CH*_4_*/CO*_2_*/N*_2_ = 40*/*40*/*100 *ccm*. Note that the material for the Spacer-2 was set MICA instead of Crofer and the reforming domain was graded with 0.088, 0.176, 0.396, 0.88 *wt*% nickel as designed in [22].

Figure 7 illustrates the comparison of the temperature profiles calculated by MICA and Crofer materials of Spacer-2. Note that the conditions are exactly the same as in the case of Figure 6. We observe that the temperature profiles in both cases are very similar along the PSC segments except the deviations around the reformer frame. We can make the same statement for ACC. However, we see that the temperature gradients are minimized within the anode when the material of the Spacer-2 was changed to MICA. In this regard, Figure 8 illustrates temperature profiles in the selected module components including the PEN components. Even if the temperature profiles of the PEN components differ in the regions which correspond to the inlet and outlet regions of the reformer, they are almost the same within anode, electrolyte, cathode. This means that by selecting a material that has a similar thermal conductivity to that of PEN components, we achieved minimizing the temperature gradients in the PEN components.

**Figure 9:**
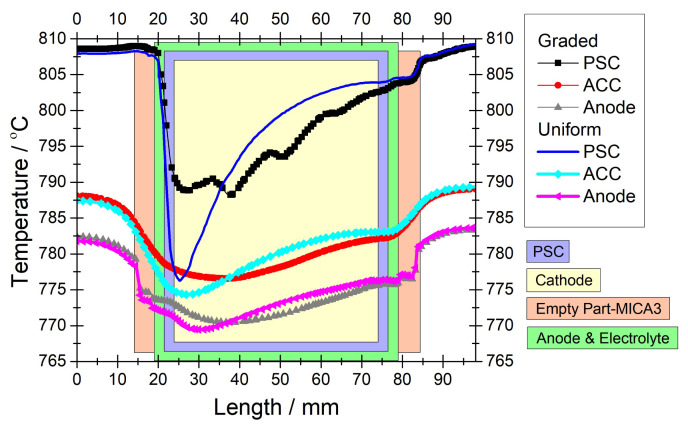
Numerical temperature profiles over various module components compared with respect to whether the reforming domain was graded. Note that the data were extracted in the middle of the reforming domain (y = 24 mm) in the flow direction for 0.3 *Acm*^−2^ and *CH*_4_*/CO*_2_*/N*_2_ = 40*/*40*/*100 *ccm*. The material for the Spacer-2 was set MICA instead of Crofer. The reforming domain was uniform with 0.22 *wt*% and it was graded with 0.088, 0.176, 0.396, 0.88 *wt*% nickel as designed in [22].

We also investigated the case if we did not grade the reforming domain at all, i.e. we had an identical nickel loading, but the PEN components were surrounded by MICA. Figure 9 compares temperature profiles of the selected module components in terms of whether the reforming domain was graded considering that the material of the Spacer-2 was MICA. We see a dramatic difference between the temperature profiles of the reforming domain and a very steep temperature drop in the case of uniform reforming domain. Even if this steep temperature drop exerts impacts on the temperature profiles of the other module components, we see that the anode exhibits similar temperature profiles in both uniform and graded cases. It can be seen that the temperature gradient in the anode was minimized, so that the risk of thermal stresses was totally eliminated.

## 4. Conclusion

In this paper, we developed a numerical model for a module of solid oxide fuel cell (SOFC). The module consisted of a single SOFC and an indirect internal reformer for utilizing biogas. In order to prevent the variation in the reforming rate, the reformer was graded in terms of catalyst loading. We analyzed the temperature distributions over the module’s components and came to the following conclusions:

- The impact of the endothermic reforming reaction on the temperature profiles of the SOFC components is limited due to the other components located among the reforming and SOFC domains. This agrees well with the concept of indirect internal reforming.
- Graded reforming domain cannot alone resolve the thermal stress issue in the indirect internal reforming SOFCs due to the fact that the mismatch among the thermal conductivities of the adjacent module components is the major reason.
- When the mismatch between thermal conductivities of the adjacent module components is eliminated, the risk of thermal stresses on the SOFC components disappears even if the reforming domain is not graded.
- For designing direct/indirect internal reforming SOFC stacks, in-depth thermal analysis of the stack is necessary for preventing development of thermal stresses on the SOFC components.

## Figures and Tables

**Table 1:** Viscosity and diffusion volume values of the reactant and product gases. Note that 1073 K was considered for calculating the viscosity of the species defined as a function of temperature.

Species	Viscosity(10^7^*Pa.s*)	Diffusion volume
*CH*_4_	103 [24]	30 [26]
*CO*_2_	170 [24]	26.7 [26]
*CO*	90 [24]	18 [26]
*H*_2_	46.96 + 0.156*T* [27]	7.07 [25]
*N*_2_	114.5 + 0.371*T* [27]	17.9 [25]
*O*_2_	101.93 + 0.306*T* [27]	16.6 [25]
*H*_2_ *O*	−9.88 + 0.361*T* [27]	12.55 [25]

**Table 2:** Specific heats of the reactant and product gases. Note that constant specific heats were computed at 1073 K.

Species	Specific heat (*J/kgK*)
*CH*_4_	((47.964(*T/*1000)^0^) + (−178.59(*T/*1000)^1^) + (712.55(*T/*1000)^2^) + (−1068.7(*T/*1000)^3^)
	+(856.93(*T/*1000)^4^) + (−358.75(*T/*1000)^5^) + (61.321(*T/*1000)^6^))*/M_CH_*__4__ [27]
*CO*_2_	((4.3669(*T/*1000)^0^) + (204.6(*T/*1000)^1^) + (−471.33(*T/*1000)^2^) + (657.88(*T/*1000)^3^)
	+(−519.9(*T/*1000)^4^) + (214.58(*T/*1000)^5^) + (−35.992(*T/*1000)^6^))*/M_CO_*__2__ [27]
*CO*	((30.429(*T/*1000)^0^) + (−8.1781(*T/*1000)^1^) + (5.2062(*T/*1000)^2^) + (41.974(*T/*1000)^3^)
	+(−66.346(*T/*1000)^4^) + (37.756(*T/*1000)^5^) + (−7.6538(*T/*1000)^6^))*/M_CO_* [27]
*H*_2_	12986 + 5.421*T* − 0.0045*T*^2^ [28]
*N*_2_	1070 − 0.198*T* + 0.00034*T*^2^ [28]
*O*_2_	896 + 0.0115*T* + 0.00026*T*^2^ [28]
*H*_2_ *O*	1672 + 0.477*T* + 0.00019*T*^2^ [28]

**Table 3:** Thermal conductivity values of the reactant and product gases. Note that 1073 K was considered for calculating the thermal conductivity of the species defined as a function of temperature.

Species	Thermal conductivity (*W/mK*)
*CH*_4_	(−23.35 + 0.1698*T* + 1.893 *×* 10^−5^*T*^2^) *×* 10^−3^ [29]
*CO*_2_	(−2.400 + 2.16 *×* 10^−2^*T* − 3.244 *×* 10^−6^*T*^2^)*/*10^5^(1004.184) [30]
*CO*	0.07379 [31]
*H*_2_	0.0784 + 3.7310^−4^*T* [28]
*N*_2_	0.0116 + 5.3910^−5^*T* [28]
*O*_2_	−0.0085 + 6.310^−5^*T* [28]
*H*_2_ *O*	−0.00784 + 8.3710^−5^*T* [28]

**Table 4:** Electrochemical properties of the SOFC electrodes.

Activation energy of anode	140 *kJ/mol* [32]
Activation energy of cathode	137 *kJ/mol*[32]
Reaction coefficient for anode	10^11^ Ω^−1^*m*^−2^ (fitted)
Reaction coefficient for cathode	10^11^ Ω^−1^*m*^−2^ (fitted)
Specific surface area of anode	2.9 *×* 10^6^ *m*^2^*/m*^3^ (fitted)
Specific surface area of cathode	2.9 *×* 10^6^ *m*^2^*/m*^3^ (fitted)

**Table 5:** Properties of the solid module components.

Specific heat of anode	450 *J/kgK* [33]
Specific heat of cathode	430 *J/kgK* [33]
Specific heat of electrolyte	470 *J/kgK* [33]
Specific heat of Crofer	660 *J/kgK* (VDM Metals GmbH, Werdohl , Germany)
Specific heat of MICA	866 *J/kgK* (Cogebi, Lot, Belgium)
Thermal conductivity of anode	1.86 *W/mK* [33]
Thermal conductivity of cathode	5.86 *W/mK*[33]
Thermal conductivity of electrolyte	2.16 *W/mK*[33]
Thermal conductivity of Crofer	24 *W/mK* (VDM Metals GmbH)
Thermal conductivity of MICA	0.22 *W/mK* (Okabe Mica Co. Ltd., Tokyo, Japan)
Density of anode	3310 *kg/m*^3^ [33]
Density of cathode	3030 *kg/m*^3^ [33]
Density of electrolyte	5160 *kg/m*^3^ [33]
Density of Crofer	7700 *kg/m*^3^ (VDM Metals GmbH)
Density of MICA	2100 *kg/m*^3^ (Okabe Mica Co. Ltd.)
Porosity of anode and cathode	0.4
Porosity of PSCs	0.9
Permeability of anode	10^−9^ *m*^2^
Permeability of cathode	10^−9^ *m*^2^
